# Langerhans Cells, T Cells, and B Cells in Oral Lichen Planus and Oral Leukoplakia

**DOI:** 10.1155/2022/5430309

**Published:** 2022-03-22

**Authors:** Amal Dafar, Angelica Siarov, Yasaman Mostaghimi, Jairo Robledo-Sierra, Shahin De Lara, Daniel Giglio, Göran Kjeller, Paulo Henrique Braz-Silva, Jenny Öhman, Bengt Hasséus

**Affiliations:** ^1^Department of Oral Medicine and Pathology, Institute of Odontology, The Sahlgrenska Academy, University of Gothenburg, Gothenburg 40530, Sweden; ^2^Department of Oral and Maxillofacial Surgery, King Fahad General Hospital, Jeddah 23325, Saudi Arabia; ^3^Faculty of Dentistry, CES University, Medellin 050021, Colombia; ^4^Department of Clinical Pathology, Sahlgrenska University Hospital, Gothenburg 41345, Sweden; ^5^Department of Oncology, Institute of Clinical Sciences, The Sahlgrenska Academy, University of Gothenburg, Gothenburg 41345, Sweden; ^6^Department of Pharmacology, Institute of Neuroscience and Physiology, The Sahlgrenska Academy, University of Gothenburg, Gothenburg 40530, Sweden; ^7^Department of Oral and Maxillofacial Surgery, The Sahlgrenska Academy, University of Gothenburg, Gothenburg 40530, Sweden; ^8^Department of Stomatology, School of Dentistry, University of Sao Paulo, Sao Paulo, Brazil; ^9^Laboratory of Virology, Institute of Tropical Medicine of Sao Paulo, School of Medicine, University of Sao Paulo, Sao Paulo 05403-000, Brazil; ^10^Clinic of Oral Medicine, Public Dental Service, Gothenburg 40233, Sweden

## Abstract

Although oral lichen planus (OLP) and oral leukoplakia (LPL) have different pathogenetic profiles, both may involve chronic inflammation. The aim of this observational study was to evaluate the inflammatory cell profiles of OLP and LPL. The inflammatory cell infiltrates in patients with OLP and LPL were analyzed for the presence of Langerhans cells (LCs; CD1a), T cells (CD3), and B cells (CD20), as well as for the proliferation marker Ki-67. Biopsied specimens from patients with OLP (*N* = 14) and LPL without dysplasia (*N* = 13) were immunohistochemically stained with antibodies directed against CD1a, CD3, CD20, and Ki-67, followed by quantitative analyses. A significant increase in the number of CD3^+^ cells and CD20^+^ cells was found in the submucosa of OLP, as compared to LPL (*p* < 0.01). Likewise, the number of CD3^+^ cells was significantly higher in the epithelium of OLP than of LPL (*p* < 0.05). No differences were found in the expression of Ki-67 and the number of CD1a^+^ cells between the two groups. Although an immune response is elicited in both conditions, there are differences at the cellular level between OLP and LPL. A more robust immune activation involving T cells and B cells is seen in OLP. The role of B cells in OLP needs to be further elucidated. Although the number of B cells in LPL is low, their role in the inflammatory response cannot be ruled out.

## 1. Introduction

Oral lichen planus (OLP) and leukoplakia (LPL) are both potentially malignant oral disorders (OPMDs), albeit with different etiologies [1]. While OLP is an autoimmune disease in which putative autoantigens in the basal cell layer trigger the inflammation, LPL probably results from genetic aberrations in keratinocytes that can elicit an immune response [2, 3].

OLP lesions are clinically bilateral, symmetrical, and present with slender white lines (Wickham's striae), which characterize the typical reticular form of OLP [4]. However, papular, plaque-like, erythematous/atrophic, ulcerative/erosive, and bullous forms of OLP are also observed [5]. The histological criteria for OLP include the presence of a band-like lymphocytic inflammatory infiltrate in the connective tissue, hydropic degeneration and apoptosis of the basal keratinocytes, and the absence of epithelial dysplasia [6]. The inflammatory cell infiltrate in OLP contains numerous CD4^+^ and CD8^+^ T cells, both in the connective tissue and in the epithelium [7]. The types of antigens that elicit CD4^+^ and CD8^+^ T-cell activation in OLP are unknown. However, dendritic Langerhans cells (LCs) have been shown to play a pivotal role in antigen presentation to T cells in OLP [8]. The malignant potential of OLP remains a matter of debate. However, chronic inflammation *per se* provides a cytokine-rich microenvironment, which may influence cell survival, growth, proliferation, differentiation, and movement, thereby contributing to cancer initiation, progression, invasion, and metastasis [2]. A recent meta-analysis reported the combined malignant transformation rate of OLP as 1.14% [9].

LPL is defined as “predominantly white plaques of questionable risk having excluded (other) known diseases or disorders that carry no increased risk of cancer” [1]. LPL is also classified as an OPMD, and the overall malignant transformation rate is 3.5% [10]. While the precise etiology of LPL is not known, genetic aberrations are most certainly involved [11], and the use of tobacco products and consumption of alcohol have been suggested as exogenous risk factors [12]. Based on its clinical appearance, LPL can be classified as homogeneous or nonhomogeneous [13]. In LPL, different histopathological findings, ranging from benign hyperkeratosis to dysplasia, are found [13]. In recent years, there has been a growing interest in understanding the immunopathogenic mechanisms of LPL by analyzing the inflammatory cell infiltrate. Öhman and co-workers were among the first to show the presence of immunosurveillance in LPL [3]. Other studies have assessed the infiltration of immune cells, such as dendritic cells, T cells, macrophages, natural killer (NK) cells, and B cells, in patients with LPL [14–16]. The malignant transformation of LPL is enigmatic and can appear either in the lesion area or elsewhere in the oral cavity, which is consistent with the “field cancerization” phenomenon [17].

An increased number of proliferating cells can be observed in tissues with chronic inflammation as well as in tissues displaying hyperplasia in comparison with healthy tissues. Indices using the proliferation marker Ki-67 have been utilized in studies of LPL [18, 19] but also OLP [20, 21]. Because OLP and LPL present with different stages of chronic inflammation and altered epithelia, Ki-67 is a biomarker that can be used to compare cell proliferation rates in the two disorders.

Although OLP and LPL have distinct pathogenetic pathways, they share the characteristic of chronic inflammation and subsequent immune activation. Thus, the aim of this observational study was to characterize and compare the presence of cells associated with immune activation such as LCs, T cells and B cells in OLP and LPL.

## 2. Materials and Methods

### 2.1. Participants

Patients who were referred to the Clinic of Oral Medicine, Public Dental Service, Gothenburg, Sweden, for suspected OLP or LPL were invited to participate in the study. The inclusion criterion was the clinical diagnosis of each condition. OLP was diagnosed clinically when multiple lesions with a symmetrical distribution were seen, commonly in the presence of Wickham's striae [22]. Different clinical subtypes of OLP are listed in Table 1. LPL was diagnosed clinically when a “predominantly white plaque of questionable risk having excluded (other) known diseases or disorders that carry no increased risk of cancer” was observed [1]. Exclusion criteria were age <18 years or the inability to understand and to read Swedish. Other exclusion criteria were the clinical diagnosis of other oral lichenoid lesions and oral fungal-infection (assessed using PAS-staining, PAS; Histolab, Gothenburg, Sweden) or the presence of dysplasia histopathologically. Written informed consent was obtained from all patients prior to the start of the study, which was conducted in accordance with Declaration of Helsinki. The Ethical Review Board in Gothenburg, Sweden, approved the study (Dnr. 618–05).

### 2.2. Data Collection

**Figure 1:**
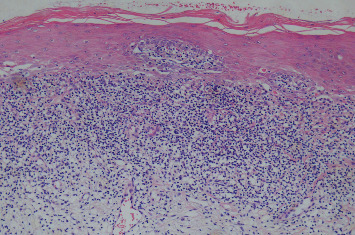
Lichenoid tissue reaction. Histopathologic examination shows a thickened surface layer of parakeratin, hydropic degeneration, apoptotic keratinocytes, and dense band-like inflammatory cell infiltrate in the superficial lamina propria. (H and E stain, Magnification x100). H and E; hematoxylin and eosin.

**Figure 2:**
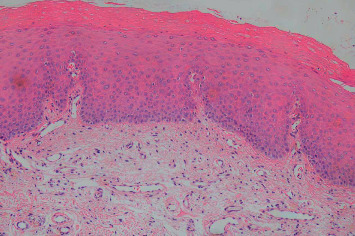
Benign hyperkeratosis with no dysplasia. Histopathologic examination shows hyperortho- and parakeratosis of stratified squamous epithelium. Mild architecture changes with acanthosis and no cellular atypia. (H and E stain, Magnification x100). H and E; hematoxylin and eosin.

For each participant, the medical history including tobacco habits, history of the oral mucosal lesion, and clinical data, including photographs, were obtained. Biopsies were taken from a total of 27 patients diagnosed clinically with OLP (*N* = 14) and LPL (*N* = 13) by oral medicine specialists. The specimens were formalin-fixed, dehydrated, and then paraffin-embedded. Sections of 4-*μ*m thickness were mounted on microscope slides; prior to deparaffinization, the sections were incubated at 60 °C for 1 h. Deparaffinization was made and the specimens on slides were oxidized with periodic acid–Schiff (PAS; Histolab, Gothenburg, Sweden). Periodic acid 1% was added for 4 min and slides were rinsed in distilled water for 5 min. The slides were then treated with the Schiff reagent for 8 min and rinsed in distilled water for 10 min. Thereafter, Mayer´s hematoxylin was used to counterstain the slides for 1 min and the slides were then rinsed with distilled water for 5 min. Thereafter, the slides were dehydrated with xylene then ethanol and finally mounted with a cover glass and mounting media. An experienced oral pathologist examined and diagnosed the specimens as lichenoid tissue reaction (Figure 1) or benign hyperkeratosis with no dysplasia (Figure 2). Furthermore, the exclusion of fungal infection was confirmed.

### 2.3. Immunohistochemistry

Anti-CD1a (ready to use, clone 010; Dako A/S, Glostrup, Denmark), anti-CD3 (ready to use, polyclonal rabbit anti-human, IR503/IS503; Dako), anti-CD20 (ready to use, clone L26; Dako), and anti-Ki67 (ready to use, clone MIB-1; Dako) antibodies were used for the immunohistochemical analyses. Sections from tonsil specimens were used as positive controls, while omission of the primary antibodies served as negative controls.

Tissue specimens were evaluated to identify LCs (CD1a), T cells (CD3), B cells (CD20), and the presence of the cell proliferation marker Ki-67 using the corresponding antibodies. Briefly, formalin-fixed, paraffin-embedded sections of 4-*μ*m thickness were mounted on microscope slides (Ref K8020; Dako). Prior to deparaffinization, the sections were incubated at 60°C for 1 h. After deparaffinization and rehydration using the DAKO PTLink system (Dako, Carpinteria, CA), antigen retrieval was carried out with the EnVision FLEX High pH Link Kit (Ref K8000, Dako) at 85°C and 97°C for 20 min each. Then, the sections were cooled down to 75°C. For immunohistochemistry, automated DAKO Autostainer Plus (Dako) was used. The sections were incubated with the antibodies for 20 min at room temperature. In order to block endogenous peroxidase activity, the EnVision FLEX Peroxidase-Blocking Reagent (Dako) was applied for 5 min. Subsequent washings were carried out with phosphate-buffered saline (PBS). The sections were then incubated with the EnVision FLEX horseradish peroxidase (SM802; Dako) for 20 min and rinsed twice in PBS. EnVision FLEX DAB + Chromogen (DM827; Dako) was added twice 5 min each time, with PBS washings in-between, to visualize the antibodies. The sections were counterstained with EnVision FLEX Hematoxylin (Ref 8018; Dako), before washing and dehydrating in a 99%–70% ethanol series and xylene for 14 min, respectively. Mountex (Histolab) was used to mount the slides.

### 2.4. Quantitative Analysis

The stained slides were examined under a light microscope at 100× magnification (Leitz Wetzlar; Leica Microsystems GmbH, Wetzlar, Germany). Two to three high-power fields (HPFs) in the lateral and central parts of sections were selected. Images of the sections were acquired with a digital camera (UC30; Olympus Sverige AB, Solna, Sweden). Positively stained nucleated cells in the epithelium and in the inflammatory cell infiltrate in the submucosa were counted manually in the digitalized images (expressed as numbers of positive cells per mm^2^) using the BioPix iQ 2.0 computer software (BioPix AB, Gothenburg, Sweden).

### 2.5. Statistical Analyses

Fischer′s exact test was used to compare the gender, presence of underlying systemic diseases, and allergies as well as the use of tobacco between patients with OLP and LPL. The Mann–Whitney *U* test was used to compare the age and the numbers of stained cells between the two groups. Statistical analyses were performed using GraphPad Prism ver. 8.0 for Mac OSX (GraphPad Software, La Jolla, CA, USA). The threshold for statistical significance was set at *p* < 0.05.

## 3. Results

### 3.1. Patients' Characteristics

Overall, 50% (7/14) of the patients with OLP and 61% (8/13) of the patients with LPL reported having systemic medical conditions. Neither OLP nor LPL patients were on systemic immunosuppressive medications. With regard to allergies, three patients with OLP reported pollen allergy and one patient reported grass and wasp sting allergy. In contrast, no allergy was reported among patients with LPL. Furthermore, 14% (2/14) of the patients with OLP and 38% (5/13) of the patients with LPL reported cigarette smoking. The corresponding values for use of the Swedish snuff (snus) were 14% (2/14) and 15% (2/13). Patients' characteristics are described in Table 1. No significant differences were found in gender, age, presence of underlying systemic diseases, and allergies as well as the use of tobacco between the patients with OLP and LPL.

### 3.2. CD1a-Expressing LCs

**Figure 3:**
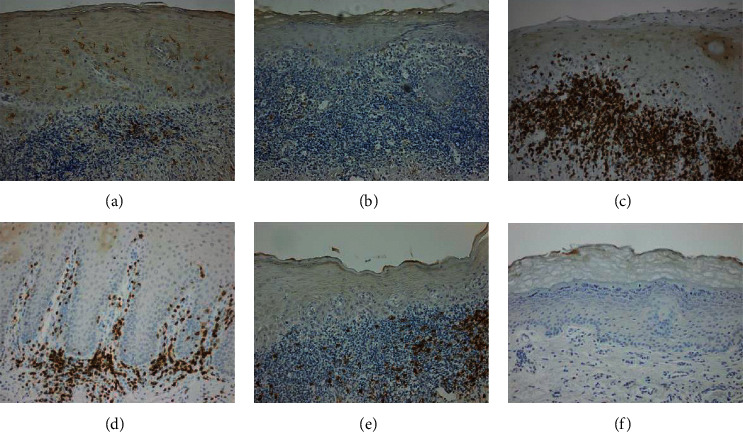
Light micrographs of: CD1a^+^ cells in (a) oral lichen planus (OLP) and (b) oral leukoplakia (LPL); CD3^+^ cells in (c) OLP and (d) LPL; CD20^+^ cells in (e) OLP and (f) LPL. Positive cells are stained brown. Magnification ×100.

**Figure 4:**
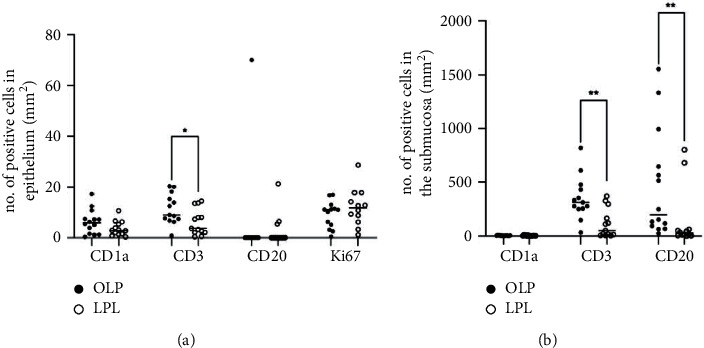
Numbers of cells expressing CD1a, CD3, CD20, and Ki67 in (a) epithelium and (b) submucosa of patients with oral lichen planus (OLP; closed circles) and leukoplakia (LPL; opened circles). Each symbol denotes a patient and the horizontal bars denote the median values. ^*∗*^*p* < 0.05; ^*∗∗*^*p* < 0.01 using the Mann–Whitney (U)-test.

CD1a^+^ LCs were detected in both the epithelium and submucosa of patients with OLP (Figure 3(a)) and patients with LPL (Figure 3(b)). The median number of CD1a^+^ LCs in the OLP epithelium was 5.8 cells/mm^2^ (range, 0.3–17.3), and in the LPL epithelium the median number of LCs was 2.8 cells/mm^2^ (range, 0.3–10.6), representing a nonsignificant difference between the groups (*p*=0.13; Figure 4(a)). Comparing the presence of LCs in the submucosal compartments of the OLP and LPL groups, the median values were 4.3 cells/mm^2^ (range, 2.4–8.5) and 2.8 cells/mm^2^ (range, 0.7–12.9), respectively, which is not a statistically significant difference (*p*=0.09; Figure 4(b)).

### 3.3. CD3-Expressing T cells

CD3^+^ cells were detected in both the epithelium and submucosa of the OLP and LPL groups (Figure 3(c) and 3(d)). In the OLP epithelium, the median number of CD3^+^ T cells was 8.9 cells/mm^2^ (range, 0.7–20.3), while in LPL epithelium the corresponding number was 3.6 cells/mm^2^ (range, 0.4–14.5). Thus, there was a higher number of T cells in the OLP epithelium than in the LPL epithelium (*p*=0.03; Figure 4(a)). In the submucosa, the median number of CD3^+^ T cells in the OLP group was 310.6 cells/mm^2^ (range, 33.2–819.4), which was significantly higher than the median of 50.2 cells/mm^2^ (range, 3.8–368.5) detected in the submucosa of the LPL group (*p*=0.0023; Figure 4(b)).

### 3.4. CD20-Expressing B cells

CD20^+^ B cells were rarely detected in the epithelium of either the OLP or LPL groups (Figure 4(a)), although they were commonly found in the submucosa (Figure 3(e) and 3(f)). In the OLP group, an outlier was seen with a number of CD20^+^ B cells in the epithelium (Figure 4(a)). Review of the histological sections of the biopsy acquired from this patient did not uncover any reason for exclusion. In the submucosa of the OLP group, 201.7 CD20^+^ B cells/mm^2^ (median number, range, 24.5–1556.1) were present, while the corresponding number for CD20^+^ B cells in the submucosa of the LPL group was 26.8 cells/mm^2^ (range, 1.1–802.5, *p*=0.0020; Figure 4(b)).

### 3.5. Ki-67 Expression

The median number of Ki-67 expressing cells was 11.0 cells/mm^2^ (range, 0.4–16.9) in the OLP epithelium and 11.7 cells/mm^2^ (range, 1.0–28.5) in the LPL epithelium (*p*=0.39; Figure 4(a)).

## 4. Discussion

The present study shows that OLP and LPL exhibit different inflammatory profiles. Increased numbers of T cells and B cells were observed in the submucosa of the patients with OLP, in comparison to the patients with LPL. In the epithelium of the patients with OLP, increased expression of CD3^+^ T cells was also found. OLP and LPL both elicit an immune response. In OLP, the inflammatory infiltrate is characterised by T cells localized in a band-like zone located beneath the epithelial basement membrane. Increased numbers of LCs are found in both the epithelium and connective tissues [23]. While the presence of B cells in OLP tissues has been studied less intensively, reports are at hand that reveal the influx of B cells and plasma cells in OLP lesions [24, 25]. The inflammatory infiltrate in LPL is usually not so prominent and shows substantial variation between patients [3]. Thus, while T cells and dendritic cells are definitely present, there are few cells belonging to the B-cell lineage. The importance of the immune system in preventing tumor development has been highlighted in several studies over the past decades [26]. Because both OLP and LPL have malignant potential, characterization of the immune profiles of these two conditions is of interest.

In the present study, we identified an increased number of CD1a^+^ LCs in the epithelium and submucosa of patients with OLP compared to LPL. The number of CD1a^+^ LCs in our study was also higher than the previously reported numbers for healthy oral mucosa [23]. Our results of the higher number of epithelial LCs in OLP compared to LPL are in line with a previous study [27]. Dendritic LCs have been attributed an important role in the initiation of the inflammatory process in OLP, possibly related to their capture and presentation of antigens to T cells [28]. It has been suggested that the increased number of LCs in the epithelium of OLP lesions is due to the capture of an unidentified antigen, leading to the mobilization of numerous LCs to the site, which in turn prevents, to some extent, the destruction of the epithelium in OLP [29]. The presence of CD1a^+^ LCs in the connective tissue may be explained by the fact that after antigen capture, the activated LCs, in an intermediate stage of maturation, migrate to the regional lymph nodes and present captured antigens to T cells [30]. In LPL, the role of LCs may be to scavenge antigens released by aberrant keratinocytes and initiate an immune response. In the present study, we saw no difference in the numbers of CD1a^+^ cells between OLP and LPL. Although the evidence is not yet conclusive, we speculate that LCs may play a role in the initiation of the immune response in both OLP and LPL.

This study also shows that CD3^+^ T cells are significantly increased in number in patients with OLP, as compared with patients with LPL. This is not surprising, as OLP is a T-cell-mediated disease [31]. Autoantigen presentation by professional antigen-presenting cells, such as LCs, may activate CD4^+^ cells, leading to the activation of cytotoxic CD8 cells, which induces apoptosis of basal keratinocytes [31]. The CD8^+^ T cells in OLP are also involved in the infiltration and destruction of the lamina propria, as well as recognition of major histocompatibility complex (MHC) I on keratinocytes as part of the process to initiate apoptosis [7, 31]. In patients with LPL, we noticed the presence of CD3^+^ cells in the epithelium and submucosa, which is in line with the findings of a previous study [3]. Öhman *et al.* have shown that CD3^+^ cells are present in the epithelium and the connective tissue of LPL lesions with or without dysplasia. However, more CD3^+^ cells were detected in dysplastic LPL [3]. Significantly fewer CD3^+^ cells were found in LPL lesions that eventually transformed to oral squamous cell carcinoma (OSCC) [15]. Accordingly, differences in the T-cell profiles of the inflammatory infiltrates between LPL and OLP may be of importance for the immunosurveillance of cell dysplasia. In this study, we chose to include LPL patients with a histopathological diagnosis of benign hyperkeratosis and without dysplasia. Dysplasia can be present in LPL but is a rare finding in OLP [32]. Dysplasia may affect the T cell profile in the inflammatory infiltrate [3]. Because the comparison in this study is between LPL and OLP, a histopathological diagnosis of benign hyperkeratosis or lichenoid reaction in both groups eliminates dysplasia as a possible confounding factor.

Only a few studies have evaluated the expression levels of CD20 in OLP and LPL [24, 33]. CD20 is expressed at different stages of B-cell maturation, including in pre-B cells, immature and mature B cells, and activated B cells, but not in plasma cells [34]. Recently, it has been shown that the expression of CD20^+^ cells is significantly higher in OLP lesions, as compared to the oral lesions of patients with acute and chronic graft-versus-host disease (GVHD) and the normal oral mucosa [35]. Nearly, 75% of atrophic OLP cases show the presence of CD20-expressing B cells [24]. The current study shows that CD20 molecules are mainly expressed on cells in the submucosal cellular infiltrate and their numbers are significantly higher in OLP than in LPL. This suggests that B cells may play an important role in maintaining the inflammation seen in OLP. In premalignant and malignant disorders, Gannot et al. have shown that the proportions of B cells (CD19 + 20) were significantly different in hyperkeratotic and mild dysplastic lesions, as compared with moderate and severe dysplastic lesions and OSCC lesions [33]. Contrary to the results of the previous study, CD19^+^ cells have been found to be significantly reduced in patients with OSCC, as compared to those with dysplastic LPL [14] suggesting that the number of CD19^+^ cells could be a major factor in the progression of LPL to OSCC. The results of our present study do not point toward a major role for at least CD20^+^ B cells in the immune response caused by LPL. It is noteworthy that none of the LPL patients in the present study had cell dysplasia. To exclude B-cell involvement, more extensive mapping of the B-cell lineage is needed.

The current study assesses cell proliferation using Ki-67 expression levels, which did not differ between the OLP and LPL epithelia. An important reason for this may be that no cases of LPL with dysplasia were included in this study.

Fungal infection of the oral epithelium may cause the recruitment of inflammatory cells, and fungi are a common finding in the LPL epithelium [36]. In our patients, negative PAS-staining excluded the possibility that fungi were influencing the inflammatory infiltrates.

## 5. Conclusions

In conclusion, the present study demonstrates that submucosal infiltration of T and B cells is more prominent in OLP than in LPL, while an immunological response is definitely present in LPL as well. This indicates that OLP is consistent with a more robust immune activation than LPL. The role of B cells in OLP needs to be further elucidated. Although the number of B cells in LPL seems to be low, a role of B cells in the inflammatory response cannot be completely ruled out.

## 6. Limitations

A limitation of the present study is the relatively small sample subsets. This limits the possibility to draw a robust conclusion. Another drawback is that the use of CD20 as the only B-cell marker excludes other B-cell maturation stages. There is a lack of knowledge regarding the influence of B cells and plasma cells on the pathogenesis of both OLP and LPL, which warrants future with more extensive studies.

## Figures and Tables

**Table 1:** Demographic and clinical characteristics of the study population.

	Oral lichen planus (OLP; *N* = 14)	Oral leukoplakia (LPL; *N* = 13)
Females/Males	8/6	6/7
Age in years (mean; median)	51 (51.5)	57.4 (57.5)
Clinical subtypes	Reticular; *N* = 7	Homogeneous; *N* = 6
	Erythematous; *N* = 5	Nonhomogeneous (nodular); *N* = 6
	Ulcerative; *N* = 1	Not specified; *N* = 1
	Papillary; *N* = 1	

Biopsy sites	Buccal mucosa; *N* = 12	Tongue; *N* = 4
	Gingiva; *N* = 1	Buccal mucosa; *N* = 3
	Mucobuccal vestibule; *N* = 1	Floor of the mouth; *N* = 3
		Lower labial mucosa; *N* = 1
		Gingiva; *N* = 1
		Palatal mucosa; *N* = 1

Histopathological diagnosis	Lichenoid reaction; *N* = 14	Benign hyperkeratosis; *N* = 11
		Lichenoid reaction; *N* = 2

Systemic diseases (%)	7 (50%)	8 (62%)
Cardiovascular disorders	3	5
Autoimmune disorders^*∗*^	3	0
Diabetes mellitus type 2	1	2
Skin disorders^*∗∗*^	3	0
Respiratory disorders	0	1
Others	1	3
Allergy	4 (28.5%)	0
Tobacco use		
Cigarette smoking (%)	2 (14.3%)	5 (38.5%)
Swedish snuff (%)	2 (14.3%)	2 (15.4%)

^*∗*^Hypothyroidism, Crohn´s disease, psoriatic arthritis. ^*∗∗*^Psoriasis, eczema.

## Data Availability

The data used to support the findings of this study can be made available from the corresponding author upon request.

## Abbreviations:

LCs: Langerhans cells
LPL: Oral leukoplakia
OLP: Oral lichen planus.
